# Board Faultlines, Innovation Strategy Decisions, and Faultline Activation: Research on Technology-Intensive Enterprises in Chinese A-Share Companies

**DOI:** 10.3389/fpsyg.2022.855610

**Published:** 2022-03-31

**Authors:** Yan Zhang, Lianfu Ma

**Affiliations:** ^1^Research Center for Environment and Sustainable Development of the China Civil Aviation, Civil Aviation University of China, Tianjin, China; ^2^Business School, Nankai University, Tianjin, China

**Keywords:** board faultlines, innovation strategy decision, social norm, dual chairman and CEO, board ownership, faultline activation

## Abstract

In the context of social norms, based on faultline theory, using samples of Chinese A-share listed companies of technology-intensive industries from 2009 to 2015, this paper studies how board faultlines influence innovation strategy decisions and test the influences of a dual chairman/CEO and board ownership on that relationship. The results of the study are as follows. Social-related faultlines have a significant negative influence on innovation strategy decisions. Cognitive-related faultlines have a significant positive influence on innovation strategy decisions. A dual chairman/CEO has no moderating effect between social-related faultlines and innovation strategy decisions, but weakens the positive effect between cognitive-related faultlines and innovation strategy decisions. Board ownership weakens the negative effect between social-related faultlines and innovation strategy decisions but enhances the positive effect between cognitive-related faultlines and innovation strategy decisions.

## Introduction

China’s 13th 5-Year Plan put forward the implementation of an innovation-driven development strategy. In this context, as the main implementers of innovation, enterprises pay more attention to the formulation of their own innovation strategy and promote the realization of national goals by realizing their own innovation goals. However, the degrees of concern of Chinese listed companies in innovation strategy are quite different. For example, in 2015, for research and development (R&D) in Chinese technology-intensive enterprises, the highest investment by companies accounted for 21.70% of their total capital, and the proportion of investment by companies that invested the least was only 0.02%. The cause of this situation has aroused deep discussion in academic circles. From the perspective of corporate governance, the board of directors plays a core role (Zahra and Pearce, 1989; Li, 2005). It is concerned with how different organizational forms and governance mechanisms affect organizational decision-making (Fama and Jensen, 2000). Existing research has proved that decision-making is an important function of the board of directors, and innovation strategy decisions are an important component of company decision-making. However, the existing research does not explain the following. First, for technology-intensive companies, boards of directors, with the same or similar features, composition, and structure, have shown great differences in their innovation strategies. Second, some companies with better overall performance and better innovation performance have different characteristics, composition, and structure. These problems challenge the decision-making of boards of directors from the perspective of board characteristics, composition, and structure. The above realistic problems enlighten academia to explore the innovation strategy decisions of boards of directors from other perspectives.

A board of directors is a special kind of group. Board decision-making is a specific manifestation of group decision-making. In the process of decision-making, the members of the board need to communicate deeply and obtain sufficient information. The interaction between members has a great influence on decision-making. Therefore, it is necessary to change the traditional research on the decisions of boards of directors to research on the decision-making process and behavior, and to discuss the process and mechanism of board decision-making. The group faultlines can be used as the basis for understanding and studying the composition and effectiveness of group diversity. It has good application in revealing the dynamic behavior of the members of the group (Lau and Murnighan, 1998). Based on previous studies on the definition, cause, and classification of board faultlines, this paper focuses on exploring the specific path of the impact of board faultlines on decision-making behavior.

This paper puts forward the path of mutual attraction, identification, exclusion, and prejudice due to board members’ social characteristics, as well as the path of information exchange, sharing, and cooperation due to the board members’ differences in cognitive ability. These two paths have an impact on communication and resource acquisition in the decision-making process of a board of directors, and affects the outcome of board decisions. This paper takes China’s technology-intensive listed companies as the research object, deeply studies board faultlines formed by the dynamic synergy effect of combined board member characteristics, verifies the two paths of board faultlines affecting the decision-making behavior and its impact on the decision results, and explores the moderating role of a dual chairman/CEO and board ownership.

## Basic Theory and Research Hypotheses

### Formation, Concept, and Connotation of Board Faultlines

Research on board faultlines stems from the research predicament of diversity and heterogeneity of boards of directors. The diversity and heterogeneity of boards of directors mainly refers to differences in gender, age, race, career and professional background, personality, and values of the members. However, domestic and foreign scholars often fail to agree or even contradict a large number of conclusions on the diversity and heterogeneity of boards of directors. For example, in their studies of board heterogeneity and corporate value creation, Carter et al. (2007); Miller and Triana (2009) show that the gender and ethnicity of board members have a positive impact on company value creation. Studies by Van der Walt and Ingley (2003) show that there is no significant correlation between the differences in gender, race, age, and other aspects of board members and the value creation of the company (Rose, 2007). This is because research on the diversity and heterogeneity of boards of directors based on demographic characteristics is only around a single feature, ignoring the other characteristics of the members and the dynamic synergy (Lau and Murnighan, 1998). Group faultlines are group differentiations caused by the combination of diverse characteristics of the team members. This concept has become a new perspective for the diversification of research teams and has attracted much attention in recent years. Lau and Murnighan give a definition of group faultlines, which is based on one or more features of the team members, dividing the team into a number of subteams by a set of imaginary cutoff lines (Lau and Murnighan, 1998). Therefore, board faultlines can be regarded as cutoff lines that divide boards into subteams. Any subteam has similar internal characteristics and different characteristics among them. Because each subteam has different behavior characteristics, in the process of the board’s internal activities, the interaction of subteams leads to communication, divergence, alienation, or contradiction.

### Board Faultlines and Corporate Innovation Strategy Decisions

Innovation strategy refers to the overall planning and action of enterprises to implement various kinds of innovation activities, usually involving the improvement and innovation of products or services (Carpenter and Westphal, 2000). The board of directors, as the core of corporate governance, plays an important role in the allocation of strategic resources, the supply of creative thinking, and the establishment of links with the outside world (Johnson et al., 2011). The traditional upper echelon theory holds that the enterprise decision-maker is the key to determining strategic decisions and the success or failure of the enterprise. The demographic characteristics and heterogeneity are important factors affecting the strategic decisions of the enterprise. The core of the theory is that the characteristics of decision-makers reflect their cognition and affect their decision-making (Hambrick, 2007). That is to say, the innovation strategy decisions of enterprises are related to the characteristics of the board of directors.

According to the concept and connotation of board faultlines, the purpose of research on board faultlines is to divide board members by a combined characteristics index, and then study the characteristics of different subteams, the process of behavior, and the results. Therefore, how to choose a combination of characteristics to form different types of board faultlines has become the key of research.

Hutzschenreuter and Horstkotte (2013) believe that group faultlines can be divided into task-related and physiological characteristic faultlines through the work-related and physiological characteristics of team members. The study also shows that these two kinds of faultlines have an impact on expansion strategy decisions of enterprises. Li and Zhou (2014) divide group faultlines into structural and cognitive dimensions according to the legal source and different cognitive characteristics of board members. Molleman (2005) divides group faultlines into shallow and deep faultlines through demographic characteristics, ability, and personality indices. Choi and Sy (2010) divide group faultlines into social-class and task-related faultlines from the point of view of team conflict. The research suggests that the mechanisms of social-class and task-related faultlines are different within the team. The two types of faultlines are related to conflicts of relations and of tasks. The research on faultlines around the word shows that the existing research method is to divide faultlines according to the combination characteristic index, the mechanism of different types of faultlines, and the different results of behavior. Therefore, studying of the influence of board faultlines on the innovation strategy of corporations in China, this paper aims to divide board faultlines into social-related and cognitive-related faultlines according to the two dimensions of the social classification and cognitive ability of the members of the board of directors.

#### Social-Related Faultlines and Corporate Innovation Strategy Decisions

Social-related faultlines are formed by characteristics such as the age, sex, nationality, or race of the members of the board of directors, which can be perceived directly by social groups and hardly change. When the board of directors carries out strategic decision-making, its members need to communicate with one another about innovative ideas and real-time information, and the social category of the board will affect the strategic decisions of the company in two aspects. First, according to the relevant research of social psychology, the cognition, attitudes, and emotions of team members with regard to other members are derived from dominant social characteristics. According to social classification and social identity theory, individuals compare themselves with other individuals to produce self-examination and self-evaluation. When individuals find similar characteristics to other individuals, this will lead to differences between the “inside group” and the “outside group” Individuals will show a strong sense of identity among the inside group and exclude members of the outside group (Messick and Massie, 1989; Phillips and A O’Reilly, 1998). Social-related faultlines will affect the interactions among the members, resulting in prejudice and discrimination among the subteams of the board of directors and impeding the process of innovative strategy decision-making. Second, the similarity attraction paradigm can also explain the formation of social-related faultlines from another perspective. According to this paradigm, similar individuals can form strong attractions and promote communication and interaction among themselves, while individual differences reduce the attraction, resulting in less communication and interaction (Hambrick, 2007). At the same time, the more similar characteristics there are between individuals, the higher the degree of communication in subteams and the more obvious faultlines between subteams. It can be seen that the existence of social-related faultlines will divide the board into subteams with different social characteristics. The greater the difference between the subteams, the deeper the extent of faultlines, which leads to a lack of communication and interaction among the subteams, as well as prejudice and discrimination, which is not conducive to the company’s innovation strategy decisions. To sum up, this paper puts forward the following hypothesis:

H1: Social-related faultlines have a negative impact on corporate innovation strategy decisions.

#### Cognitive-Related Faultlines and Corporate Innovation Strategy Decisions

Cognitive-related faultlines refers to faultlines caused by differences in knowledge and views due to differences in professional skills, knowledge background, and functional background of the board members (Tuggle, 2010; Li and Zhou, 2014). The higher the diversity of the board members in terms of professional skills, knowledge background, and functional background, the more abundant the professional knowledge and professional perspective, and it has a positive effect on the corporate innovation strategy. When making strategic decisions, members of the board of directors will have to deal with different types of information and data. The existence of cognitive-related faultlines will help board members understand and absorb different types of market information and help them make innovative strategy decisions. Based on the hypothesis of cognitive diversity, Williams and O’Reilly believe that cognitive diversity can bring advantages to team processes and output, including creativity, quality of decision-making, and the ability to solve problems (Williams and O’Reilly, 1998). The differences between the members of the board of directors in professional skills, knowledge background, and functional background will help to create innovative ideas and avoid “group thinking” in the process of group decision-making. At the same time, the cognitive information processing perspective can also explain cognitive-related faultlines formed by the board members based on different cognitive abilities, which makes the members have different perceptions on the problem of the company’s innovation strategy and hold different views on how to make decisions (Li and Zhou, 2014). Cognitive-related faultlines increase the information value of the board members, which is beneficial to the flow, exchange, and sharing of knowledge and information among them and is conducive to the formation of high-quality and innovative strategy decisions. To sum up, this paper puts forward the following hypothesis:

H2: Cognitive-related faultlines have a positive impact on corporate innovation strategy decisions.

### Faultline Activation

Lau and Murnighan (1998) first proposed the concept of group faultline activation in their research. This and other studies suggest that there are a number of potential cutoff lines within the group that do not work at all times, but will be activated in a particular situation. For example, when the group is discussing the problem of retirement, the group faultlines formed by the age characteristics of the director will be activated. In the same way, when companies are facing problems such as introducing and distributing scarce resources, the group faultlines formed by the functional characteristics of the directors will be activated. On the basis of Lau’s study, Karen and Katerina (2010) explicitly proposed two concepts, “potential faultlines” and “faultline activation”. Bezrukova et al. (2010) believe that the individual members of a group are aware of the differences in individual characteristics under the influence of certain circumstances or factors, and then form the division of the group. This process from differentiation to group division is the activation process of group faultlines. Fan and Du (2015) further elaborated that the potential group faultlines are only the objective existence of an internal division of the group, which does not have a practical impact on the group. However, when group faultlines are activated, they will affect the group behavior or decision-making and organizational performance. Then, on the basis of many existing studies, Du (2016) proposed the “activation effectiveness of faultlines,” which mainly describes the difficulty of various factors to activate group faultlines.

#### Activation of Board Faultlines by a Dual Chairman/CEO

Having a dual chairman/CEO is a common situation in modern enterprises. According to principal-agent theory, the manager of a company is a rational person whose goal of action is to maximize self-utility, which will lead to the use of company resources to make a profit. With a dual chairman/CEO, the decision-making power, executive power, control, and supervision power of the company are controlled by the same person. In this case, it will be difficult for the CEO to supervise and control the use of corporate resources for personal gain, which will lead to failure of the internal supervision mechanism. In addition, a dual chairman/CEO will also lead to failure of the internal control mechanism in corporate governance. In this case, the CEO will use his or her own power to intervene in the decisions of other members of the board, and to press or interfere with the decision-making of other members through personal influence in the discussion of the issues of board meetings, and to destroy the control ability of the board of directors.

As to the influence on the relationship between board faultlines and decision-making, a dual chairman/CEO makes the chairman’s power more centralized in the company and affects the degree of differentiation and conflict among the subteams formed by board faultlines. Of course, it may also lead the chairman to achieve his or her personal purpose. In addition, traditional Chinese culture has the idea of “distinction between noble and humble, respect for seniority,” which has a profound impact on Chinese corporate governance. Therefore, within the board of directors, the chairman with the greatest power will profoundly affect the cognitive preferences and choices of other directors and control their decision-making behavior. Thus, other directors who are in different subteams with the chairman are also easily influenced or controlled by the authority of the chairman.

An enterprise’s innovation strategy decision-making is an important kind of decision-making, with high uncertainty and great information demand. The decision-makers are more likely to have varied opinions and queries. According to the aforementioned theoretical analysis, a dual chairman/CEO will cause two kinds of situations inside the company. First, the chairman of the board will have greater power, greater authority, and greater voice in the company. Second, the enterprise’s decision-makers are more likely to form shared goals, disperse decision-making responsibility, and promote the formation of risk decision.

In the innovation strategy decisions of the company, social-related faultlines are mainly based on social classifications and the similarity-attraction paradigm, which make the board of directors have excessive conflict, contradiction, prejudice, and discrimination and hinder the formation of innovation strategy. A dual chairman/CEO leads to maximization of power, which will prompt the other members of the board to form an attitude and inclination toward loyalty and obedience. This phenomenon will weaken the negative influence of the social-related faultlines on the company’s innovation strategy decisions. The great power brought by a dual chairman/CEO will inhibit and obstruct the diverse views and opinions of the board of directors, which is not conducive to the creation of innovative ideas. In this case, cognitive-related faultlines do not promote effective communication within the board of directors and impede the flow and exchange of information. A dual chairman/CEO will destroy the positive impact of cognitive-related faultlines on the company’s innovation strategy decisions. To sum up, this paper puts forward the following hypotheses:

H3a: A dual chairman/CEO has a moderating effect on the relationship between social-related faultlines and innovation strategy decisions. Compared with separate chairman and CEO, integrating the two positions weakens the negative impact of social-related faultlines on innovation strategy decisions.

H3b: A dual chairman/CEO has a moderating effect on the relationship between cognitive-related faultlines and innovation strategy decisions. Compared with separate chairman and CEO, integrating the two positions weakens the positive impact of cognitive-related faultlines on innovation strategy decisions.

#### Activation of Board Faultlines by Board Ownership

The separation of ownership and management of the modern company leads to differences between managers and owners in the interests and position of the company, and the two are different from the goal of the company. The principal-agent theory holds that equity incentive is one of the main ways to solve the inconsistency between the interests of managers and owners. Board ownership can make the interests of managers and owners align, and encourage managers to pay more attention to the long-term development of the company. At the same time, board ownership can transfer part of the control to the managers, that is, the directors and executives. This will encourage the managers to reach their personal potential and tap the potential of the company through continuous innovation and change to realize maximum value for the company’s shareholders.

Board ownership can make directors have a certain shareholder identity. If the share of the board of directors is large, directors and shareholders will have a shared goal. The shared goal of the subteams will make them more consistent with the company, which can lessen the conflict and contradiction caused by board faultlines. This will help the company’s directors to cooperate across the faultlines, to avoid the company’s personal interests and misconduct.

With the continuous improvement of China’s corporate governance system and capital market, a series of incentives, such as equity incentives, have been used in practice. Through incentive compatibility, the shareholders and board members of a company can form consistent judgments on the direction of the company’s operation and consistent recognition of the company’s value. From within the board of directors, there are subteams of different types and different degrees of “split” because of board faultlines, but board ownership can promote these different subteams to share a goal with shareholders. Homan et al. (2008) show that implementing effective incentive mechanisms in the diversity of teams can promote target identity among subteams and lessen the negative impact of group faultlines. The incentive mechanism has an obvious regulating effect. In addition, Kaczmareks et al. (2012) have found that the link between the executive director’s remuneration and the company’s performance can promote the commitment of the directors to the company and the shareholders. Moreover, this will help to achieve the goals of individual board members and the board as a whole for shareholders and reduce the negative impact of board faultlines.

Board ownership can realize the identity of the board of directors for the company’s goals, avoid or reduce antagonism between the subteams, and promote cooperation among board members across the cracks among the subteams. At the same time, the target identity of board ownership can promote the board of directors to actively participate in the strategic decisions of the company, determine the development potential of the company, and consider how to realize the value added of the company’s future with the shareholders. Therefore, this paper holds that raising the shareholder ratio of the board of directors can realize the consensus of the value of the company and weaken the adverse effects caused by board faultlines, and there is a regulatory role in the relationship between board faultlines and the strategic decisions of the company. To sum up, this paper puts forward the following hypotheses:

H4a: Board ownership plays a moderating role in the relationship between social-related faultlines and innovation strategy decisions. The board of directors has a relatively high shareholding ratio, which weakens the negative influence of social-related faultlines on the company’s innovation strategy decisions.

H4b: Board ownership plays a moderating role in the relationship between cognitive-related faultlines and innovation strategy decisions. The board of directors has a relatively high shareholding ratio, which strengthens the positive influence of cognitive-related faultlines on the company’s innovation strategy decisions.

**FIGURE 1:**
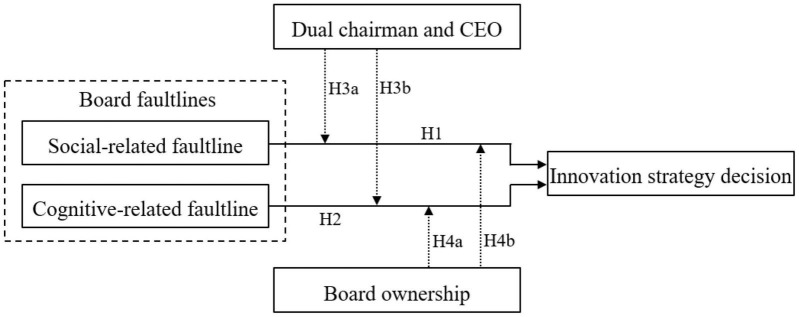
Conceptual model.

The conceptual model of this paper is shown in Figure 1.

## Research Design

### Sample Selection and Data Sources

Considering the situation in China, this paper takes Chinese technology-intensive enterprises as the object of study, referring to the division of Lu and Dang (2014). At the same time, considering the requirements of the China Securities Regulatory Commission, since 2007, Chinese listed companies have made information disclosures on R&D expenditure. Therefore, this paper selects data of 2009–2015 A-share listed companies in the electronic, machinery, equipment, instruments, pharmaceutical, biopharmaceutical, other manufacturing, and information technology industries. The company data needed for the research were collected from each company’s annual report and China stock market and accounting research database. This paper also supplements and evidences research data from authoritative media such as Sina Net, Phoenix Net, and the annual reports of listed companies with the same board members. In order to improve the rigor of research, the sample data were processed as follows:

First, we eliminated ST and *ST companies, and companies that no longer belong to the above industries after reorganization or changing their main business. Second, we eliminated companies with incomplete R&D, financial, and governance data disclosure. Third, we eliminated companies listed later than the research window period. Finally, we got 2170 samples from 310 companies in 2009–2015. In order to eliminate the influence of extreme values, this paper carries out winsorizing for continuous variables at the 1% level.

### Variables and Measures

#### Dependent Variable

Innovation strategy decision (*ISD*). Referring to the research of David (2001); Olson et al. (2006), this paper chooses the company’s innovation investment as the proxy variable for innovation strategy decision. A company’s innovation investment is mainly decided by the board of directors, which reflects the decision to allocate the resources of the innovation strategy. It is the direct result of the innovation strategic decision. There are two main types of indicators for the measurement of innovation investment (Daellenbach et al., 2002). The first is the scale of R&D investment, which is expressed in terms of the natural logarithm of R&D expenditure. The second is the intensity of R&D investment, which is measured by the proportion of R&D expenditure to operating income, total assets, or the market value of the enterprise. Because management can easily control business income, the reliability of the sample data is poor. Therefore, this paper uses the proportion of R&D expenditure to total assets to measure the level of innovation strategy decisions.

#### Independent Variables

Social-related faultline (*SRF*). According to the above analysis, social-related faultlines are mainly measured by the age, sex, race, and other characteristics of board members. However, considering the small racial differences in the sample selected in this study, racial traits are not used as a measure of social class disruption.

Cognitive-related faultline (*CRF*). According to Pelled (1996), members having different professional backgrounds and educational levels in a diverse team can produce knowledge collisions and integration. Therefore, this paper selects professional background and education level to measure cognitive-related faultlines.

The measurement method for social-related and cognitive-related faultlines is based on Lau’s method (Lau and Murnighan, 1998). According to Lau’s study, group faultlines are measured using a bisection pattern, which divides the group into two subgroups according to the criteria. The reason is that when the group size is small, it is hard to divide it into three or more subgroups (Thatcher et al., 2003). Therefore, the equation for calculating *SRF* and *CRF* is as follows:


(1) $$Fau_{\text{g}}=\frac{\sum_{j=1}^{p}\sum_{k=1}^{2}n_{k}^{\text{g}}{\left({\bar{x}}_{jk}-{\bar{x}}_{j}\right)}^{2}}{\sum_{j=1}^{p}\sum_{k=1}^{2}\sum_{i=1}^{n_{k}^{\text{g}}}{\left(x_{ijk}-{\bar{x}}_{j}\right)}^{2}}$$

*g = 1, 2, 3, …, S*

For a board of directors with *n* members, the classification of faultlines is 2*^n^*^–1^−1. In Eq. (1), *n* stands for the number of members on the board; *p* stands for the total number of features examined; *g* stands for the classification; _$n_{k}^{g}$_ represents the number of members in subteam *k*, which is classified by way of *g*; _${\bar{x}}_{j}$_ represents the average value of all board members on characteristic *j*; _${\bar{x}}_{\mathrm{jk}}$_ represents the average value of members in subteam *k* on characteristic *j*; *x*_*ijk*_ represents the value of member *i* on characteristic *j* in subteam *k*; *Fau*_*g*_ is the degree of board faultlines under the *g* classification and is between 0 and 1. The larger the value, the stronger the faultlines, and vice versa.

#### Regulating Variables

##### Dual Chairman and CEO (*Dual*)

As an active factor to investigate the impact of board faultlines on the innovation strategy decisions of the company, the existing research methods are more consistent with the variables, and most scholars choose the method of dummy variable. When the CEO is also the chairman of the board of directors, *Dual* = 1. In this situation, the CEO has greater power over and influence on the board of directors. When the CEO and chairman are not one person, *Dual* = 0. This is contrary to the previous situation.

##### Board Ownership (*Bstock*)

One of the most fundamental problems of corporate governance is the principal-agent problem. Board ownership can effectively solve the agency problem between shareholders and executives, and promote the two entities to form a consistent corporate goal. As an active factor to investigate the impact of board faultlines on the innovation strategy decisions of the company, to measure board ownership, academia mainly uses the method of board ownership ratio, as shown in Eq. (2):


(2) $$\begin{array}{c} Boardownershipratio=Boardownership/Allsharesofthe\\ company\times 100\% \end{array}$$

#### Control Variables

Earnings of the previous year (*ROA*_*t*–1_). The earnings of the previous year will have an impact on enterprise strategy.

##### Company Size (*Size*)

This study holds that the size of the company is directly proportional to the resources it owns. The greater the company size, the more support and security can be provided for innovation. This paper uses total company capital to measure company size.

##### Size of the Board of Directors (*Bsize*)

To a certain extent, this variable reflects the diversity of the board members’ background. It may have an impact on the company’s innovation strategy decisions. This paper takes it as a control variable and measures it using the number of board members.

##### Company Growth Ability (*Growth*)

According to the existing research, the company’s growth ability will also affect its innovation strategy. Andriopoulos and Lewis (2009) believe that innovation is positively related to future growth ability. Companies with high growth are more concerned about innovation and are more inclined to invest in innovation. Therefore, this paper chooses growth ability as the control variable and takes it into the research model. The company growth ability is measured by the growth rate of the main business revenue.

##### Ownership Concentration (*Herf*)

According to the existing research, the main components and forces of the company’s major decisions have an important impact on its innovation decisions. In view of this, this paper considers the proportion of the largest shareholder (*Herf1*) and the second to 10th largest shareholders (*Herf2–10*) as the measure of ownership concentration. This paper chooses the Herfindahl index method to calculate, and the calculation method of this index is shown in Eq. (3):


(3) $$H\left(n\right)=\Sigma_{i=1}^{n}\theta {}^{2}$$

In Eq. (3), *H*(*n*) stands for the degree of ownership concentration of the former *n* major shareholders, θ^2^ stands for the square of shareholding ratio of shareholder *i*, and $\Sigma_{i=1}^{n}$ stands for the square sum of the former *n* shareholder’s shareholding ratio. When *H*(*n*) = 1, it means that all shares of the company are concentrated in the hands of the former *n* major shareholders. The larger the Herfindahl index, the more concentrated the company’s stock in the former *n* shareholders.

##### Asset Liability Ratio (*Leverage*)

According to the relevant research, the company’s liabilities will have an impact on its operation. When the debt level of the company is high, creditors may have an impact, controlling or interfering in the company’s decision-making. Comparatively speaking, when the company’s debt level is relatively low, it is not easy for the creditors to interfere in or influence the company’s decision-making. Therefore, this study selects the company’s asset liability ratio as the control variable, and the calculation method is shown in Eq. (4):


(4) $$\begin{array}{c} Assetliabilityratio=Totalliabilities/totalassetsofthe\\ company\times 100\% \end{array}$$

Length of establishment of the company (*Age*). This research holds that the company’s innovation strategy decisions are related to the life cycle of the enterprise. The development stage of the company will have an impact on its innovation strategy decisions. This paper uses the length of establishment of the company to measure it.

The dependent variables, independent variables, regulating variables, and control variables are shown in Table 1.

**TABLE 1:** Main variables.

Variable type	Abbreviation	Variable name	Variable description
**Dependent**	*ISD*	Innovation strategy decision	Proportion of R&D cost to total assets
**Independent**	*SRF*	Social-related faultline	Investigation of age and sex characteristics
	*CRF*	Cognitive-related faultline	Investigation of professional background and educational level
**Regulating**	*Dual*	Dual chairman/CEO	Whether the chairman is CEO
	*Bstock*	Board ownership	Degree of shareholding of board of directors
**Control**	*ROA_*t*–1_*	Earnings of the previous year	Company’s earnings in the previous year
	*Size*	Company size	Total company capital
	*Bsize*	Size of the board of directors	Members of the board at the end of the year
	*Growth*	Company growth ability	Growth rate of the company’s main business income
	*Herf1*	Proportion of largest shareholder	Using the Herfindahl index
	*Herf2–10*	Proportions of second to 10th largest shareholders	Using the Herfindahl index
	*Leverage*	Asset liability ratio	Ratio of total liabilities to total amount of company assets
	*Age*	Length of establishment of the company	Time of company’s establishment

### Model Setting

In order to test the function mechanism of social-related and cognitive-related faultlines on the company’s innovation strategy decisions, the activation mechanism of dual chairman/CEO, and board ownership to board faultlines, this paper establishes the following two research models to test the hypotheses, as shown in Eqs. (5, 6):


(5) ISD=α+β IndependentiVariables+γΣjControlVariables+ε

(6) ISD=α+β Independent Variablesi+δ IndependentiVariables×Micro+γΣjControlVariables+ε

Among them, *ISD* is the dependent variable, representing the company’s innovation strategy decisions; *Independent Variables* represents social-related and cognitive-related faultlines; Σ*Control Variables* represents the control variables; *Micro* represents the regulating variable^1^; β_*i*_ is the coefficient of the explanatory variable; γ_*i*_ is the coefficient of the control variable; δ_*i*_ is the coefficient of the interaction term; α is the intercept term; and ε is the residual term.

## Data Analysis

### Descriptive Statistics and Correlation Test

The descriptive statistics show that the mean value of *ISD* is 0.0218 with a standard deviation of 0.0254; the mean value of *SRF* is 0.5346 with a standard deviation of 0.0586; the mean value of *CRF* is 0.2258 with a standard deviation of 0.0906. Testing the correlation coefficient of the main variables of the sample firms shows that there are two related relationships, between *SRF* and *ISD* and between *CRF* and *ISD*. The correlation between independent variables is not more than 0.4. It is proved that there is no serious multiple collinearity among the variables in the study model, which can be further studied. The correlation coefficient matrix of the main variables is shown in Table 2.

**TABLE 2:** Correlations coefficient of each variable.

Variable	1	2	3	4	5	6
(1) *ISD*	1					
(2) *SRF*	−0.093**	1				
(3) *CRF*	0.076***	−0.072*	1			
(4) *Bstock*	0.074**	−0.015*	0.021**	1		
(5) *ROA_*t*–1_*	−0.026**	−0.027*	0.027***	0.170	1	
(6) *Size*	0.056***	0.040***	−0.047**	0.039**	0.005**	1
(7) *Bsize*	0.016**	0.014**	−0.015**	−0.028**	0.047**	0.061***
(8) *Growth*	−0.074***	0.065**	0.007*	−0.315*	−0.297*	0.047**
(9) *Herf1*	−0.038**	−0.082**	0.092**	0.098*	0.156**	0.075**
(10) *Herf2–10*	−0.043*	−0.092*	−0.001*	0.104*	0.132*	0.128**
(11) *Leverage*	0.001***	−0.049**	0.087**	−0.103*	0.057**	−0.089*
(12) *Age*	0.130***	0.142**	0.143**	0.021**	0.005	−0.056***

**Variable**	**7**	**8**	**9**	**10**	**11**	**12**

(7) *Bsize*	1					
(8) *Growth*	0.061***	1				
(9) *Herf1*	0.129***	0.120**	1			
(10) *Herf2–10*	0.100**	0.101*	0.277**	1		
(11) *Leverage*	−0.127**	0.054**	0.182***	0.209**	1	
(12) *Age*	−0.074***	0.028**	−0.314*	−0.299***	0.153**	1

*Significant at 10% level; **significant at 5% level; ***significant at 1% level.

### Regression Analysis

This paper uses multiple linear regression in Stata 14.0 software to analyze the relationship between social-related and cognitive-related faultlines and innovation strategy decisions. This paper also tests the influence of dual chairman/CEO and board ownership on the above relationships. Considering the possible heteroscedasticity of sample data, this paper uses ordinary least square regression of robust standard deviation modified to test the hypotheses. The results are detailed in Table 3, in which model 1 only carries out regression analysis on control variables and innovative strategy decisions, and models 2 and 3 add *SRF* and *CRF* to the regression test on the basis of model 1. The variance inflation factor (*VIF*) of all variables in the model was lower than 2, with an average value of 1.33, which was significantly lower than the critical value of 10.0 recommended by Neter et al. (1999). It is proved again that there is no multicollinearity in variables selected in this study. Model 2 shows that there is a significant negative correlation between social-related faultline (*SRF*) (_β_1__ = –0.394, *p* < 0.01) and innovation strategy decision (*ISD*); model 3 shows that there is a significant positive correlation between cognitive-related faultline (*CRF*) (_β_2__ = 0.209, *p* < 0.01) and innovation strategic decision (*ISD*). Thus, H1 and H2 were verified in this study.

**TABLE 3:** Board faultlines and innovation strategy decisions.

Variable	Model 1	Model 2	Model 3
*SRF*		–0.394***	
		(–3.45)	
*CRF*			0.209***
			(2.41)
*ROA_*t*–1_*	0.000***	0.000***	0.000***
	(1.33)	(1.23)	(1.42)
*Size*	–0.015**	–0.015**	–0.018**
	(–2.35)	(–2.39)	(–2.35)
*Bsize*	0.002***	0.002***	0.003***
	(0.37)	(0.35)	(0.47)
*Growth*	0.014**	0.013	0.013
	(0.51)	(0.53)	(0.50)
*Herf1*	0.000***	0.000***	0.000***
	(3.15)	(3.11)	(3.17)
*Herf2–10*	0.000***	0.000***	0.000***
	(5.68)	(5.85)	(5.82)
*Leverage*	–0.016***	–0.016***	–0.016***
	(–5.54)	(–5.47)	(–5.44)
*Age*	–0.000	–0.000	–0.000
	(–0.23)	(–0.19)	(–0.18)
*R* ^2^	0.332	0.363	0.365
*Adj-R^2^*	0.312	0.339	0.335
*F-value*	84.71***	87.33***	86.34***
*N*	2170	2170	2170

*Significant at 10% level; **significant at 5% level; ***significant at 1% level.

Models 4 and 5 are used to examine the impact of dual chairman/CEO on the relationship between social-related faultlines and innovation strategy decisions. When the group sample is *Dual* = 0, social-related faultline (*SRF*) and innovation strategic decision (*ISD*) are negative but not significant (_β_1__ = –0.123,*p* > 0.1). When the group sample is *Dual* = 1, social-related faultline (*CRF*) and innovation strategic decision (*ISD*) are still negative but not significant (_β_1__ = –0.135, *p* > 0.1). The regression results of models 4 and 5 show that no matter whether the chairman and CEO are two individuals or not, there is no significant correlation between social-related faultlines and innovation strategy decisions. Therefore, it can be concluded that dual chairman/CEO has no moderating effect on the relationship between social-related faultlines and the company’s innovation strategy decisions. Thus, H3a was not proved in this study.

Models 6 and 7 are used to examine the impact of a dual chairman/CEO on the relationship between cognitive-related faultlines and innovation strategy decisions. When the group sample is *Dual* = 0, with a confidence level of 99%, cognitive-related faultline (*CRF*) is significantly positively correlated with innovation strategy decision (*ISD*) (_β_1__ = 0.254, *p* < 0.01). When the group sample is *Dual* = 1, with a confidence level of 95%, cognitive-related faultline (*CRF*) is significantly positively correlated with innovation strategy decision (*ISD*) (_β_1__ = 0.270, *p* < 0.05). Comparing the regression results between models 6 and 7, the confidence level of the regression test is reduced from 99% to 95%. The results show that a dual chairman/CEO is a negative activator of board faultlines, which can reduce the activation of cognitive-related faultlines and have a negative influence on innovation strategy decisions. Therefore, the test of models 6 and 7 proves that a dual chairman/CEO has a moderating effect on the relationship between cognitive-related faultlines and innovation strategy decisions. A dual chairman/CEO weakens the positive impact of cognitive-related faultlines on innovation strategy decisions, as shown in Table 4.

**TABLE 4:** Impact of dual chairman/CEO on the relationship between board faultlines and innovation strategy decisions.

Variable	Model 4	Model 5	Model 6	Model 7
	(*Dual* = 0)	(*Dual* = 1)	(*Dual* = 0)	(*Dual* = 1)
*SRF*	–0.123	–0.135		
	(–2.49)	(–2.77)		
*CRF*			–0.254***	0.270**
			(3.72)	(3.97)
*ROA_*t*–1_*	0.001**	0.001***	0.001**	0.000**
	(1.20)	(1.26)	(1.17)	(1.28)
*Size*	–0.009**	–0.015**	–0.011**	–0.018**
	(–2.31)	(–2.73)	(–2.57)	(–3.03)
*Bsize*	0.003**	0.003**	0.003**	0.003***
	(1.52)	(1.67)	(1.52)	(1.67)
*Growth*	0.013*	0.015	0.036*	0.037
	(0.52)	(0.69)	(0.49)	(0.63)
*Herf1*	0.000***	0.000***	0.000***	0.000***
	(1.84)	(2.98)	(2.04)	(3.45)
*Herf2–10*	0.000**	0.000**	0.000**	0.000*
	(3.37)	(3.87)	(3.41)	(3.64)
*Leverage*	–0.014*	–0.014**	–0.016*	–0.016*
	(–5.38)	(–5.38)	(–4.98)	(–4.93)
*Age*	–0.000	–0.000	–0.000	–0.000
	(–0.29)	(–0.16)	(–0.24)	(–0.19)
*R* ^2^	0.297	0.298	0.297	0.298
*Adj-R^2^*	0.297	0.298	0.268	0.258
*F-value*	36.42***	57.21***	38.34***	59.17***
*N*	677	1493	677	1493

*Significant at 10% level; **significant at 5% level; ***significant at 1% level.

Introducing board ownership (*Bstock*) and the interaction of social-related faultlines and board ownership (SRF*Bstock) into model 2 produces model 8. Regression analysis shows that model 8 affirms the negative impact of social-related faultline (S*RF*) on innovation strategy decision (*ISD*) (_β_1__ = –0.130, *p* < 0.05). At a confidence level of 99%, model 8 also confirmed that board ownership (*Bstock*) significantly weakens the effect of social-related faultline (*SRF*) on innovation strategy decision (*ISD*) (_β_1__ = 1.131, *p* < 0.01). By a regression test of models 2 and 8, it can be verified that board ownership (*Bstock*) has a moderating effect on the relationship between social-related faultline (*SRF*) and innovation strategy decision (*ISD*). The board has a high shareholding ratio, which weakens the negative influence of social-related faultlines on innovation strategy decisions. Thus, H4a was verified in this study.

Introducing board ownership (*Bstock*) and the interaction of cognitive-related faultlines and board ownership (CRF*Bstock) into model 3 produces model 9. Regression analysis shows that model 9 affirms the positive impact of cognitive-related faultline (*CRF*) on innovation strategy decision (*ISD*) (_β_1__ = 0.357, *p* < 0.1). At a confidence level of 95%, model 9 also confirmed that board ownership (*Bstock*) significantly strengthens the effect of cognitive-related faultline (*CRF*) on innovation strategy decision (*ISD*) (_β_1__ = 0.564, *p* < 0.05). By regression test of models 3 and 9, it can be verified that board ownership (*Bstock*) has a moderating effect on the relationship between cognitive-related faultline (*CRF*) and innovation strategy decision (*ISD*). The board has a high shareholding ratio, which strengthens the positive influence of cognitive-related faultlines on innovation strategy decisions. Thus, H4b was verified in this study, as shown in Table 5.

**TABLE 5:** Impact of board ownership on the relationship between board faultlines and innovation strategy decisions.

Variable	Model 2	Model 8	Model 3	Model 9
*SRF*	–0.394***	–0.367***		
	(–3.45)	(–3.56)		
*CRF*			0.209***	0.315***
			(2.41)	(4.03)
*Bstock*		–0.130**		0.357*
		(–0.71)		(0.95)
SRF*Bstock		1.131***		
		(2.35)		
CRF*Bstock				0.564**
				(0.69)
*ROA_*t*–1_*	0.000***	0.001***	0.000***	0.001***
	(1.23)	(1.37)	(1.42)	(1.40)
*Size*	–0.015**	–0.021**	–0.018**	–0.025***
	(–2.39)	(–2.73)	(–2.35)	(–3.16)
*Bsize*	0.002***	0.003**	0.003***	0.003***
	(0.35)	(0.44)	(0.47)	(0.45)
*Growth*	0.013	0.014	0.013	0.015
	(0.53)	(0.62)	(0.50)	(0.68)
*Herf1*	0.000***	0.000***	0.000***	0.000***
	(3.11)	(2.01)	(3.17)	(2.11)
*Herf2–10*	0.000***	0.000***	0.000***	0.000***
	(5.85)	(5.87)	(5.82)	(5.84)
*Leverage*	–0.016***	–0.016**	–0.016***	–0.016***
	(–5.47)	(–5.33)	(–5.44)	(–5.51)
*Age*	–0.000	–0.000	–0.000	–0.000
	(–0.19)	(–0.22)	(–0.18)	(–0.21)
*R* ^2^	0.363	0.366	0.365	0.370
*Adj-R^2^*	0.339	0.347	0.335	0.351
*F-value*	40.16***	43.27***	86.34***	89.12***
*N*	2170	2170	2170	2170

*Significant at 10% level; **significant at 5% level; ***significant at 1% level.

### Robustness Check

In order to ensure the robustness of the research results, a robustness test was carried out. The robustness test is mainly based on two aspects of measurement variables and endogenous control.

Regarding remeasurement of the dependent variable, this study selects the degree of R&D investment as an alternative variable for innovation strategy decisions. In order to carry out the robustness test, this paper chooses two methods to measure the dependent variable again. The first method is to measure the ratio of R&D expenditure to company revenue; the second method is to measure the ratio of R&D expenditure to market value. After the regression test, it was found that the results are consistent with the conclusions obtained in this study. In this paper, the independent variables in the study are also remeasured. For social-related faultlines, this paper uses the gender and working term of directors to replace gender and age. For cognitive-related faultlines, this paper uses educational level and professional experience to replace educational level and professional background. The regression results using the new measuring method are consistent with the previous regression results.

In endogenous control, considering the possible endogeneity between independent variables and dependent variables, dealing with data in a lag stage can solve this problem. Therefore, this paper deals with board faultline data in a lag phase. Regression analysis shows that the research results are not affected. It can be seen that there is no serious endogenous problem between independent variables, dependent variables, and moderating variables.

Through a new round of analysis, it is proved that the conclusion of this study has certain robustness and reliability.

## Results and Discussion

The results of regression analysis supported H1 and H2. The greater the degree of social-related faultlines, the more serious the prejudice and discrimination between the different subteams formed by those faultlines, resulting in a lack of in-depth communication and interaction within the board of directors. This is not conducive to full analysis and discussion of strategic problems, and ultimately is not conducive to innovation strategy decisions. With a greater degree of cognitive-related faultlines, the different subteams formed by those faultlines can avoid the phenomenon of “group thinking” in decision-making. This facilitates the exchange and sharing of knowledge and information among directors, and promotes the formation of innovative strategy decisions.

The results of the regression analysis in this paper do not support H3a, although a dual chairman/CEO can effectively reduce individual responsibility in the company’s risk decision-making, which may lead to an increased possibility of innovation strategy decision-making. However, a dual chairman/CEO will also greatly increase the authority and influence of the chairman and make the board members form an attitude of respect, loyalty, and obedience, which will cause the decision behavior to be seriously affected by the chairman. In addition, a dual chairman/CEO will also facilitate decision-making and implementation of subjects with shared and consistent goals. Under the combined effect of the above factors, the moderating effect of a dual chairman/CEO on the relationship between social-related faultlines and innovation strategy decisions has not been effectively confirmed. At the same time, this study proved that H3b was established. The individual authority brought by the two positions of chairman and general manager will restrict the diversification of the board of directors and the formation of different views, which is not conducive to creative thinking by the board. At the same time, the results of regression analysis supported H3b. As the personal authority brought by a dual chairman/CEO will restrict the formation of diverse perspectives within the board of directors, it is not conducive to the activation of innovative thinking. Therefore, the negative activation mechanism of a dual chairman/CEO in cognitive-related faultlines is confirmed.

The results of regression analysis supported H4a and H4b. This study proves that board ownership can promote the company’s board of directors to form a consistent value goal. On the one hand, the value identity of convergence can weaken the negative impact of social-related faultlines on innovation strategy decisions. On the other hand, it can strengthen the positive impact of cognitive-related faultlines on innovation strategy decisions. The results demonstrate the moderating effect of board ownership on the relationship between board faultlines and innovation strategy decisions. Board ownership in social-related and cognitive-related faultlines has significant activation effects on innovation decisions.

## Conclusion

This paper verifies the relationship between board faultlines and the company’s innovation strategy decisions. The research has important theoretical and practical significance for the problem of a lack of innovation decisions and R&D investment in China’s technology-intensive enterprises. First, board faultlines becomes an important variable to study board governance after the traditional variables of board composition and diversity. At the same time, this study changes from the decision of the board of directors to the decision-making process of the board of directors, and discusses the influence of prejudice, communication, interaction, and information acquisition in the decision-making process. Second, this study examines the impact of social-related and cognitive-related faultlines on innovation strategy decisions. The research conclusions will help technology-intensive enterprises to pay more attention to board governance, promote the formation of reasonable social-related and cognitive-related faultlines, and optimize the quality of employment of the directors. Third, the two factors of dual chairman/CEO and board ownership are the internal governance environment of the company. In the practice of the company, it should consider the activation mechanism of these two factors to improve the level of its innovation strategy decisions. Therefore, on the one hand, the board of directors should carefully consider and balance the advantages and disadvantages of a dual chairman/CEO. On the other hand, on the premise of dual chairman/CEO, in order to avoid the negative activation of cognitive-related faultlines, the board of directors needs to reduce the personal power and influence of a dual chairman/CEO. In addition, the company should give full play to the positive impact of board ownership on innovation strategy decisions, and improve the board ownership ratio, such as by equity incentives, so as to promote the positive performance of innovation strategy decisions.

This research still has the following limitations. First, this research uses the proportion of R&D expenditure in the company’s total assets to measure innovation strategy decisions. However, the number and proportion of research staff and the number of patents can be considered instead of this variable. Future research can use these indicators to measure innovation strategy decisions. Second, through literature research and related theoretical analysis, this paper selects the characteristics of the directors’ age, gender, educational level, and professional background as the basis for the measurement of board faultlines. In fact, such characteristics as the value, personality, and emotions of the director can be used to measure board faultlines, but considering factors such as data acquisition, this paper does not consider other possible measures. The shortcomings and limitations of the above research will be the focus of future research.

## Data Availability Statement

The raw data supporting the conclusions of this article will be made available by the authors, without undue reservation.

## Author Contributions

YZ proposed the research questions, designed the research scheme, collected data, conducted statistical analysis, and wrote the draft of the manuscript. LM provided valuable suggestions and revised it. Both authors have approved the version of this manuscript.

## Conflict of Interest

The authors declare that the research was conducted in the absence of any commercial or financial relationships that could be construed as a potential conflict of interest.

## Publisher’s Note

All claims expressed in this article are solely those of the authors and do not necessarily represent those of their affiliated organizations, or those of the publisher, the editors and the reviewers. Any product that may be evaluated in this article, or claim that may be made by its manufacturer, is not guaranteed or endorsed by the publisher.

## Footnotes

1 Dual is tested by grouping, so there is no interaction term.
